# Epigenetic Contributions to Clinical Risk Prediction of Cardiovascular Disease

**DOI:** 10.1161/CIRCGEN.123.004265

**Published:** 2024-01-30

**Authors:** Aleksandra D. Chybowska, Danni A. Gadd, Yipeng Cheng, Elena Bernabeu, Archie Campbell, Rosie M. Walker, Andrew M. McIntosh, Nicola Wrobel, Lee Murphy, Paul Welsh, Naveed Sattar, Jackie F. Price, Daniel L. McCartney, Kathryn L. Evans, Riccardo E. Marioni

**Affiliations:** 1 Centre for Genomic and Experimental Medicine, Institute of Genetics and Cancer (A.D.C., D.A.G., Y.C., E.B., A.C., D.L.M., K.L.E., R.E.M.), The University of Edinburgh, United Kingdom.; 2 Division of Psychiatry, Royal Edinburgh Hospital (A.M.M.), The University of Edinburgh, United Kingdom.; 3 Edinburgh Clinical Research Facility, Western General Hospital (N.W., L.M.), The University of Edinburgh, United Kingdom.; 4 Usher Institute, Old Medical School (J.F.P.), The University of Edinburgh, United Kingdom.; 5 School of Psychology, University of Exeter, United Kingdom (R.M.W.).; 6 Institute of Cardiovascular and Medical Sciences, British Heart Foundation Glasgow Cardiovascular Research Centre, University of Glasgow, United Kingdom (P.W., N.S.).

**Keywords:** biomarkers, cardiovascular diseases, epigenomics, multiomics, troponin

## Abstract

**BACKGROUND:**

Cardiovascular disease (CVD) is among the leading causes of death worldwide. The discovery of new omics biomarkers could help to improve risk stratification algorithms and expand our understanding of molecular pathways contributing to the disease. Here, ASSIGN—a cardiovascular risk prediction tool recommended for use in Scotland—was examined in tandem with epigenetic and proteomic features in risk prediction models in ≥12 657 participants from the Generation Scotland cohort.

**METHODS:**

Previously generated DNA methylation–derived epigenetic scores (EpiScores) for 109 protein levels were considered, in addition to both measured levels and an EpiScore for cTnI (cardiac troponin I). The associations between individual protein EpiScores and the CVD risk were examined using Cox regression (n_cases_≥1274; n_controls_≥11 383) and visualized in a tailored R application. Splitting the cohort into independent training (n=6880) and test (n=3659) subsets, a composite CVD EpiScore was then developed.

**RESULTS:**

Sixty-five protein EpiScores were associated with incident CVD independently of ASSIGN and the measured concentration of cTnI (*P*<0.05), over a follow-up of up to 16 years of electronic health record linkage. The most significant EpiScores were for proteins involved in metabolic, immune response, and tissue development/regeneration pathways. A composite CVD EpiScore (based on 45 protein EpiScores) was a significant predictor of CVD risk independent of ASSIGN and the concentration of cTnI (hazard ratio, 1.32; *P*=3.7×10^−3^; 0.3% increase in C-statistic).

**CONCLUSIONS:**

EpiScores for circulating protein levels are associated with CVD risk independent of traditional risk factors and may increase our understanding of the etiology of the disease.

**See Editorial by Bozack et al**

For the past 20 years, cardiovascular disease (CVD) has been among the leading causes of mortality and morbidity worldwide. Given that many CVD cases are preventable, it is important to identify at-risk individuals early, when an intervention is most likely to be effective, and translate this knowledge into preventative strategies.^1,2^ Although there are many CVD risk prediction algorithms, currently, they have limited predictive performance.^3^ It may be possible to improve on that by discovering novel factors strongly associated with the disease, for example, the type and the concentrations of proteins expressed as a response to the damage to the cardiovascular system.

Several proteins have been highlighted as possible biomarkers for CVD. These include GDF15 (growth differentiation factor 15), NT-proBNP (N-terminal pro-B-type natriuretic peptide), and ADM (adrenomedullin).^4–7^ An established and highly sensitive marker of myocardial damage is cardiac troponin.^8^ It is a complex of 3 proteins, namely, cTnI (cardiac troponin I), cTnT (cardiac troponin T), and cTnC (cardiac troponin C) regulating the contraction of the cardiac muscle. Cardiac forms of troponin T^9,10^ and troponin I are expressed almost exclusively in the heart.^11^ Following myocyte damage, cardiac troponin enters the circulation and can be detected in blood samples. A high-sensitivity cardiac troponin test plays a role in the rapid diagnosis of myocardial infarction.^8^ Low-grade elevations in cardiac troponin are associated with an increased risk of CVD.^8^

Individual differences in protein concentration can be well captured by DNA methylation (DNAm). DNAm is a type of epigenetic modification characterized by the addition of methyl groups to DNA. Typically, the methyl group is added to cytosine-phosphate-guanine dinucleotides that are found mostly (but not exclusively) in gene promoters.^12^ Blocking promoters, to which activating transcription factors should bind to initiate transcription, is one of the mechanisms by which DNAm can precisely regulate gene expression.^13^ Conversely, changes in DNAm patterns can also be a result of changes in gene expression and chromatin state.^14,15^

DNAm-based proxies for protein levels are referred to as protein epigenetic scores (EpiScores) and are broadly analogous to polygenic risk scores. These methylation scores can be derived from penalized linear regression models of protein concentrations. Due to their temporal stability, protein EpiScores may exhibit stronger associations with disease outcomes than singular protein measurements, which are known to fluctuate between measurements.^16–19^ We have shown that EpiScores for 109 circulating protein levels are associated with the time to diagnosis for a host of leading causes of morbidity and mortality, including cardiovascular outcomes.^20^ Protein EpiScores are, therefore, useful biomarker tools for disease risk stratification.

Here, we examine whether protein EpiScores, calculated for ≥12 657 participants of the Generation Scotland (GS), study can augment predictions made by a CVD risk calculator developed for use in Scotland (ASSIGN^21^). We first run individual Cox proportional hazard (PH) models to discover relationships between individual protein EpiScores and incident CVD. We then create a CVD EpiScore (based on the protein EpiScores) and test the additional predictive performance offered by it for CVD risk stratification. A graphical overview of the analyses is presented in Figure 1.

**Figure 1.**
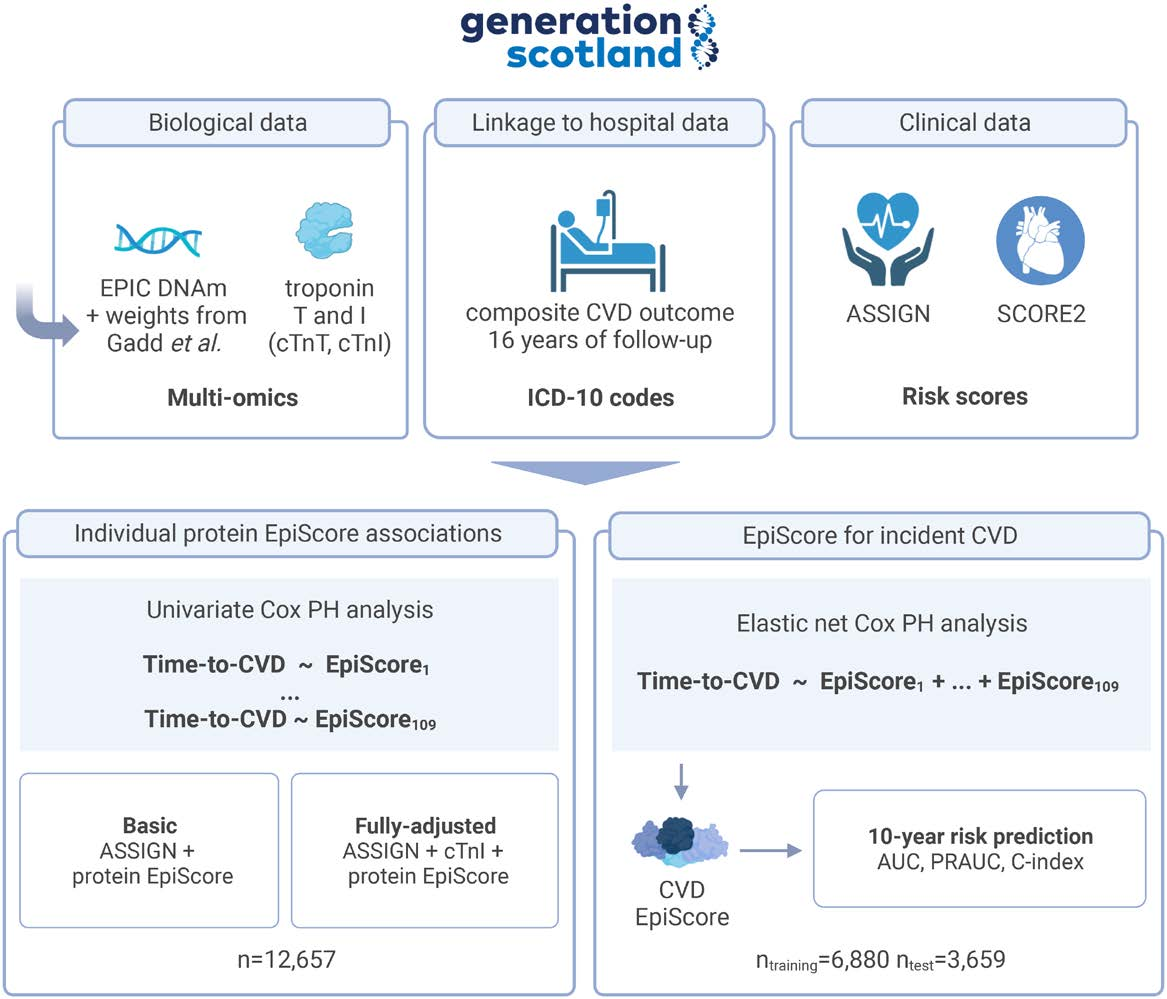
**Project overview.** A series of Cox proportional hazard (PH) models were run to model the relationship between time-to-cardiovascular disease (CVD) and 109 protein epigenetic scores (EpiScores). Basic models were adjusted for the ASSIGN score, whereas fully adjusted models also included the concentration of cTnI (cardiac troponin I). This was followed by a prediction analysis where a composite protein EpiScore was trained. The CVD EpiScore was derived using elastic net and 109 protein EpiScores as possible input features. The score was assessed in the test sample to quantify the additional predictive performance offered by it over and above ASSIGN and SCORE2. The test Cox PH models were adjusted for age, sex, cTnI, and the CVD EpiScore, with time-to-CVD as the outcome. ASSIGN indicates the cardiovascular risk score chosen for use by SIGN (Scottish Intercollegiate Guidelines Network) and Scottish Government Health Directorates; AUC, area under the receiver operating characteristic curve; cTnT, cardiac troponin T; PRAUC, area under the precision recall curve; and SCORE2, an algorithm derived, calibrated, and validated to predict 10-year risk of first-onset CVD in European populations. Created with BioRender.com

## METHODS

All methods are described in the Supplemental Material. A key resource in this study, GS, is a family-based research initiative focusing on genetic and environmental factors influencing health. Briefly, from 2006 to 2011, eligible individuals were selected from participating general medical practices in Scotland and invited at random to take part in the study.^22^ All participants provided written informed consent for research. The study received ethical approval from the National Health Service Tayside Committee on Medical Research Ethics (REC reference number: 05/S1401/89). The GS data set is not publicly available as it contains information that could compromise participant consent and confidentiality. However, the data, research materials, and analytical methods will be made accessible to other researchers for the purpose of replicating the findings. Access will be granted upon successful project application to the GS Access Committee and obtaining ethical approval for accessing linked health data from NHS Scotland. Instructions for accessing GS data can be found at https://www.ed.ac.uk/generation-scotland/for-researchers/access; the GS Access Request Form can be downloaded from this site.

## RESULTS

### Clinical Risk Prediction Tools

ASSIGN scores were calculated for 16 366 individuals with nonmissing risk factor data. To meet the PH assumption of the Cox model, the data set was filtered to individuals aged between 30 and 70 years (results split by decade are presented in Table SI) and trimmed of outliers (points beyond 3 SDs of the mean; n=181). This left a cohort of 12 790 individuals, which was further filtered to records with nonmissing concentrations of cTnI (n=12 657). Table 1 summarizes the training, test, and full data sets.

**Table 1.**
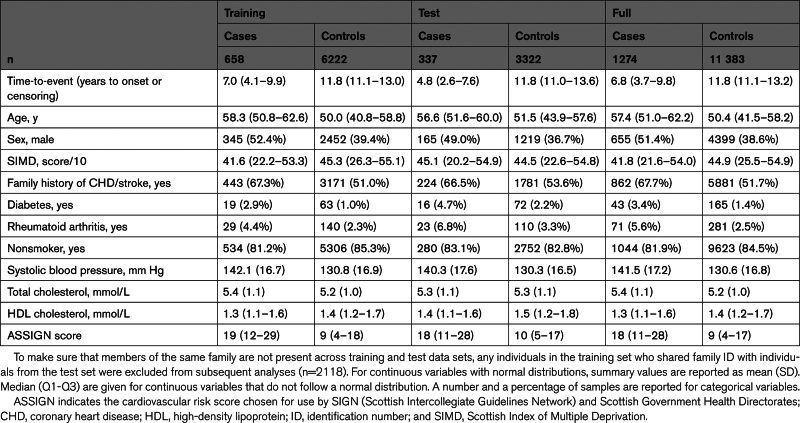
Summary of Training, Test, and Full Data Sets. The Full Data Set Contains Related Individuals

### Incremental Model Using Cardiac Troponin and Cardiac Troponin EpiScores

We tested whether concentrations of cardiac troponin were associated with CVD risk above ASSIGN over 16 years of follow-up. While the measured concentration of cTnI was associated with a hazard ratio (HR) of 1.20 per SD increase in the full (n=12 657) cohort (95% CI, 1.13–1.29; *P*=1.9×10^-8^), an EpiScore generated for cTnI (see Methods for details) was not associated with the measured concentrations in the n=3659 test set (incremental R^2^, 0.027%; *P*=0.31) and did not predict CVD risk in Cox models adjusted for ASSIGN in the same test set (*P*=0.59). For that reason, it was not considered a feature in the generation of the composite CVD score.

### Incremental Model Using EpiScores for Plasma Protein Levels

We then tested whether 109 protein EpiScores generated by Gadd et al^20^ (protein description available in Table SII) were associated with CVD risk over 16 years of follow-up (n=12 657; n_events_=1274).

First, we generated 109 Cox PH CVD risk models adjusted for ASSIGN. Each model was additionally adjusted for a different protein EpiScore. Two EpiScores failed to satisfy the PH assumption (Schoenfeld residual test *P*>0.05), and 6 EpiScores were not unique (proxied the concentration of the same protein). Of the remaining 101 protein EpiScores, 67 were significantly associated with CVD risk (*P*<0.05). After applying a conservative Bonferroni threshold for multiple testing (*P*<0.05/101=5.0×10^−4^), 36 associations remained statistically significant.

**Figure 2.**
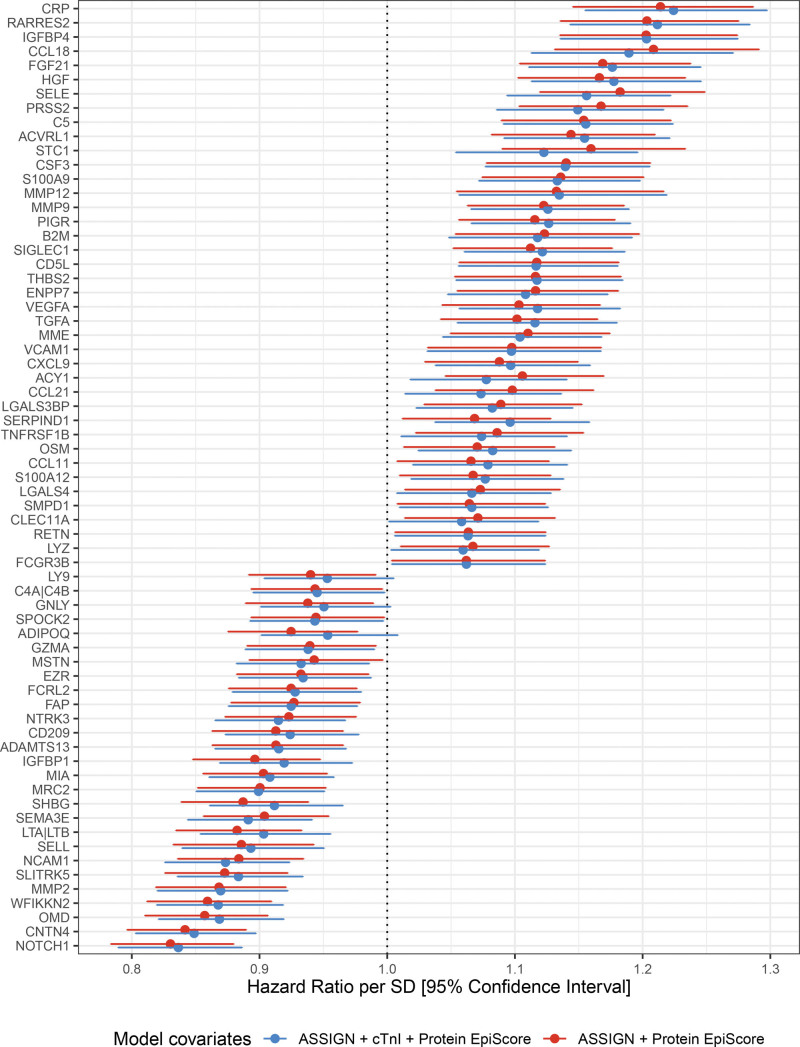
**Associations between protein epigenetic scores (EpiScores) and incident cardiovascular disease.** Hazard ratios are plotted for the 67 significant associations (P<0.05) with 95% CI limits. Basic models were adjusted for ASSIGN (red), whereas full models included the ASSIGN score and concentration of cTnI (cardiac troponin I) as covariates (blue).

**Figure 3.**
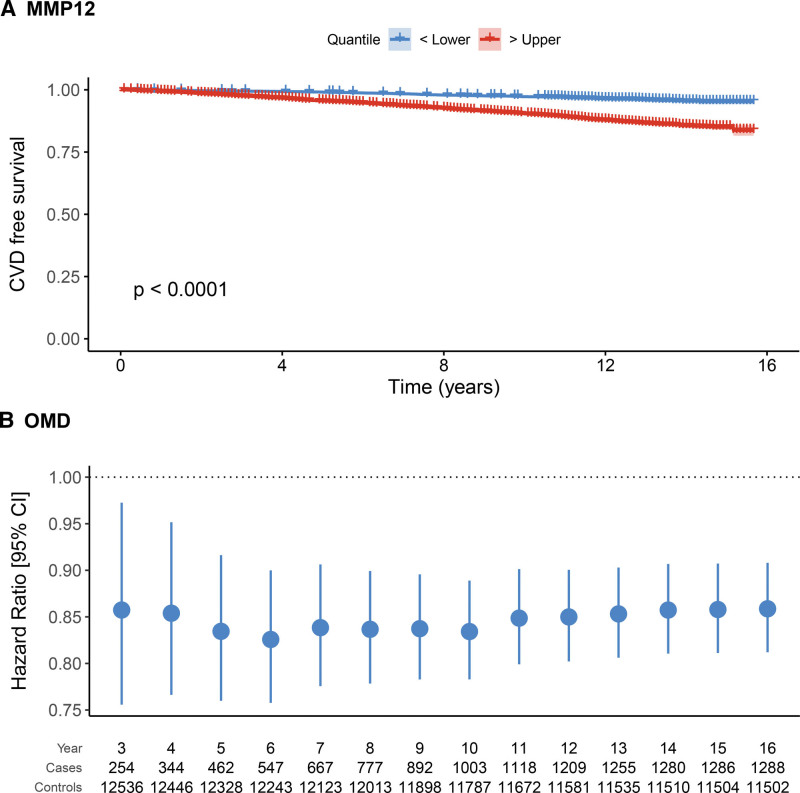
**Changes in cardiovascular disease (CVD) free survival and CVD risk plotted for two selected protein EpiScores. A**, Individuals with higher levels of MMP12 (>75th percentile) had shorter CVD-free survival when compared with those with lower levels of this EpiScore (<25th percentile). **B**, Hazard ratios (per SD of the EpiScore) and 95% CIs associated with the levels of OMD EpiScore plotted over time. At all examined time points, the association with CVD risk was significant (P<0.05).

Secondly, to understand whether protein EpiScores were associated with CVD risk beyond established biomarkers such as cardiac troponin, we included the concentration of cTnI as a covariate in the model along with ASSIGN, and we repeated the analysis. Of the 101 aforementioned protein EpiScores, 65 were associated with CVD over and above the ASSIGN score and the concentration of cTnI (*P*<0.05; Figure 2). Thirty-three associations remained significant after correcting for multiple tests. Of the 65 protein EpiScores, higher levels of 41 were associated with an increased hazard of CVD (HR>1 and *P*<0.05). For example, elevated levels of CRP and MMP12 were associated with HR per SD of 1.23 (95% CI, 1.16–1.30; *P*=9.2×10^−12^) and 1.13 (95% CI, 1.06–1.22; *P*=5.4×10^−4^; Figure 3A), respectively. In contrast, higher levels of 24 protein EpiScores were associated with a decreased hazard of CVD (HR<1 and *P*<0.05). Examples of protein EpiScores belonging to this group include NOTCH1 (HR per SD, 0.84 [95% CI, 0.79–0.89]; *P*=1.6×10^−9^) and OMD (HR per SD, 0.87 [95% CI, 0.82–0.92]; *P*=1.0×10^−6^). The relationships between individual EpiScores and CVD risk have been visualized in the form of risk-over-time (Figure 3B), forest, and Kaplan Meier plots in an online R application (https://shiny.igc.ed.ac.uk/3d2c8245001b4e67875ddf2ee3fcbad2/).

As DNAm levels vary between different types of white blood cells, there is a concern that the associations that we observe may be influenced by cellular heterogeneity. To mitigate this potential effect, we incorporated estimated white blood cell proportions as covariates in the model adjusted for the concentration of cTnI and the ASSIGN score. In this model, 50 protein EpiScores were significantly associated with CVD risk (*P*<0.05). The comparison of HRs associated with protein EpiScores in each of the studied models can be found in Table SIII.

Finally, to learn whether individual protein EpiScore can augment CVD prediction beyond established biomarkers and clinical risk prediction tools, we calculated C-statistics for null and full models. While the null model was adjusted for ASSIGN and the concentration of cTnI (C-stat, 0.728), the full model also contained the studied protein EpiScore. Table 2 lists the top 10 associations that result in the greatest improvement in CVD risk prediction.

**Table 2.**
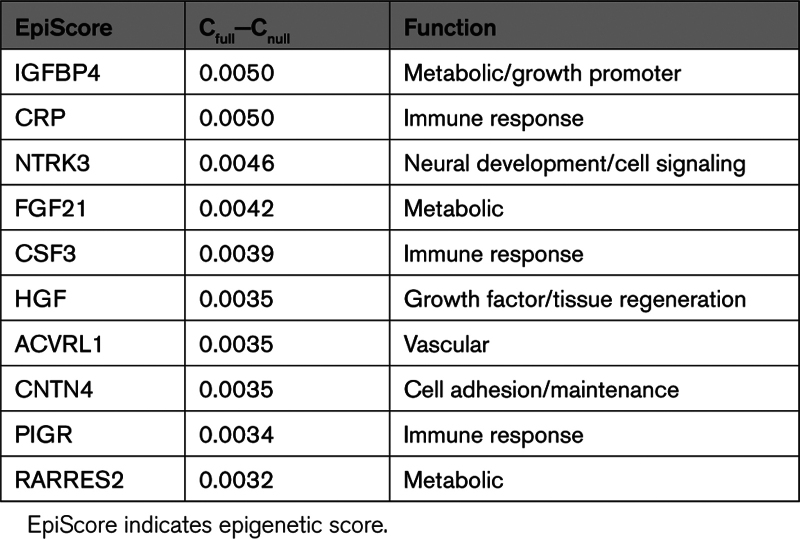
C-Statistics Calculated for Null and Full Protein EpiScore Models. Risk Was Ascertained Over 16 Years of Follow-Up. The Null Model Was Adjusted for ASSIGN and the Concentration of Cardiac Troponin I, While the Full Model Also Included a Studied EpiScore

### Composite Episcore for CVD Risk Prediction

To understand whether the abovementioned protein EpiScores can be used as biomarkers that add additional predictive value over and above typically used clinical risk scores (ASSIGN and SCORE2) and the concentration of cTnI, we generated a composite CVD EpiScore—a weighted linear combination of individual protein EpiScores. The score was trained using 2 modeling techniques: Cox PH Elastic Net and Random Survival Forest. There were 6880 records in the training set and 3659 records in the test set. The Elastic Net assigned nonzero coefficients to 45 of 109 protein EpiScores (Table SIV).

In a 10-year Elastic Net prediction analysis, the null model (containing age, sex, and ASSIGN) had an area under the receiver operating characteristic curve (AUC) of 0.719. The model with the CVD EpiScore increased the AUC to 0.723. The addition of cTnI to the null model resulted in an AUC of 0.721. The full model (null model+cTnI+CVD EpiScore) AUC was 0.724. Full output for the CVD models including C-statistics and a comparison with SCORE2 can be found in Tables V through VII. These analyses were a carbon copy of the aforementioned ASSIGN models—a null model (containing age, sex, and SCORE2) was compared with models with cTnI and the CVD EpiScore. The CVD EpiScore remained statistically significant after adjusting for the concentration of cTnI in models incorporating ASSIGN and SCORE2 (HR, 1.32; *P*=3.7×10^−3^ and HR, 1.36; *P*=1.4×10^−3^, respectively).

Random Survival Forest–based analysis (see Methods) yielded similar results. The null model (as above) had an AUC of 0.719. Adding the CVD EpiScore to the null model increased the AUC to 0.721. The full model adjusted for CVD EpiScore and the concentration of cardiac troponin had an AUC of 0.723.

## DISCUSSION

In this study, we describe 65 novel epigenetic biomarkers that are associated with long-term risk of CVD independently of a clinical risk prediction tool (ASSIGN) and the concentration of an established protein biomarker (cTnI). The most statistically significant EpiScores reflected concentrations of proteins involved in metabolic, immune, and developmental pathways. A weighted linear combination of protein EpiScores (the composite protein-CVD EpiScore) was significantly associated with CVD risk in models adjusted for ASSIGN. Although the score may be a useful addition to other omic features in future CVD risk prediction tools, at present, it is unlikely to be measured in a clinical setting.^23^

One previous study focused on how DNAm biomarkers improve CVD risk prediction.^24^ Using time-to-event data and a panel of 60 blood DNAm biomarkers measured in an Italian cohort of 1803 individuals (295 cases), Cappozzo et al^24^ trained a composite score for predicting short-term risk of CVD. In comparison, we focused on a more extensive panel of DNAm protein markers in addition to measured troponin levels. We also ran univariate analyses to identify individual proteins and protein classes that are associated with CVD. Furthermore, we developed 10-year prediction models (the prediction window for which both ASSIGN and SCORE2 are recommended) trained on more than double the number of cases.

Our findings suggest that individual protein EpiScores capture disease-specific biomarker signals relevant to CVD risk prediction. The relationships found between 65 protein EpiScores and incident CVD mirrored previously reported associations between CVD and measured protein concentrations. For example, elevated levels of CRP, a marker for systemic low-grade inflammation, have been associated with multiple age-related morbidities, including CVD.^25^ MMP12 and OMD, in turn, are involved in maintaining the stability of atherosclerotic plaques. While MMP12 contributes to the growth and destabilization of plaques,^26^ increased levels of OMD have been observed in macrocalcified plaques from asymptomatic patients.^27^ Finally, multiple studies have demonstrated that NOTCH1 signaling protects the heart from CVD-induced myocardial damage. The Notch1 pathway is involved in neoangiogenesis and revascularization of a failing heart.^28^ It limits the extent of ischemic injury,^28^ reduces fibrosis,^29^ and improves cardiac function.^30^ Several protein EpiScores associated with CVD in our study, such as SELE and C5, have also been shown to be associated with stroke and ischemic heart disease in our previous work.^20^

Whereas some of the EpiScores reflect known protein-CVD associations, others reflect novel pathways. This includes, but is not limited to, PRSS2 and CNTN4. PRSS2, which encodes the digestive enzyme trypsin 2, has been mainly studied in the context of pancreatitis. However, recent studies provide evidence that trypsin can leak from the small intestine into the bloodstream and digest myocardial tissue during heart failure.^31^ Trypsin-mediated degradation of heart tissue was also observed in cases of dilated cardiomyopathy following influenza A infection.^32^ CNTN4, in turn, encodes a cell adhesion molecule implicated in the development of autism spectrum disorders.^33^ Recent studies have shown that mutations in CNTN4 were associated with an elevated production of a prothrombotic agent called thromboxane A2 and an increased risk of cardiovascular events.^34^

The protein EpiScore that we trained for cTnI was not associated with the incidence of CVD. Therefore, we excluded it from composite CVD score generation. This highlights an important consideration in the development of multiomics biomarkers, as there are unlikely to be DNAm differences that associate with every blood protein. For example, the 109 protein EpiScores generated by Gadd et al^20^ that we make use of in our study were extracted as the best-performing EpiScores from a total set of 953 proteins tested as potential outcomes. It is, therefore, not always possible to generate a meaningful protein EpiScore that reflects the protein biology. In the case of cardiac troponins, the elevations in circulating cTnI and cTnT are a result of a leakage of these proteins from the damaged heart muscle into the bloodstream.^35^ As opposed to transcription, this process is not regulated by DNAm. Therefore, the methylation signal underlying an increased concentration of cardiac troponin in the bloodstream may be too weak to enable the generation of a meaningful EpiScore. This limitation may also extend to other proteins derived in the heart or other tissues involved in CVD onset. Nonetheless, the ability of a DNAm array to capture surrogate markers for hundreds of proteins—many of which are not routinely measured in the clinic—offers promise in the development of CVD biomarkers.

Strengths of this study include the precise timing of the CVD event through the electronic health records, the ability to generate a clinical risk predictor in a population cohort, and the large sample size for DNAm, which also permitted the splitting of the data into train/test sets to formally examine the improvement in risk prediction from our omics biomarkers.

Limitations to this work include the generalizability beyond a Scottish population. In this study, we trained and tested predictors in a Scottish cohort to augment the ASSIGN score. However, many of the protein EpiScores were trained in a German cohort (KORA [Cooperative Health Research in the Region Augsburg]) and projected to GS.^20^ This suggests that the EpiScore biomarkers part-translate across European ancestry populations. Although the ASSIGN score is tailored to the Scottish population, we observed similar findings across all models when replacing it with SCORE2, which is widely used across Europe. To generalize the findings further, replication of the EpiScore associations with CVD (while adjusting for SCORE2) across other European ancestry populations is required.

## CONCLUSIONS

In conclusion, we identified novel epigenetic signals that were associated with the incidence of CVD independently of ASSIGN and the concentration of cardiac troponin. The exploration of associations between protein EpiScores and CVD shed light on the etiology and molecular biology of the disease. As DNAm and proteins are assessed in increasingly large cohort samples, it will be possible to evaluate more precisely the potential gains in risk prediction, disease prevention, and any associated health economic benefits.

## ARTICLE INFORMATION

### Acknowledgments

The authors are grateful to all the families who took part, the general practitioners and the Scottish School of Primary Care for their help in recruiting them, and the whole Generation Scotland team, which includes interviewers, computer and laboratory technicians, clerical workers, research scientists, volunteers, managers, receptionists, health care assistants, and nurses. They would also like to thank Dr Robert Hillary for helpful feedback on the article and analyses.

### Sources of Funding

This research was funded in whole, or in part, by the Wellcome Trust (104036/Z/14/Z, 108890/Z/15/Z, 220857/Z/20/Z, and 216767/Z/19/Z). For the purpose of open access, the author has applied a CC BY public copyright license to any author-accepted manuscript version arising from this submission. A.D. Chybowska was supported by a Medical Research Council PhD Studentship in Precision Medicine with funding from the Medical Research Council Doctoral Training Program and the University of Edinburgh College of Medicine and Veterinary Medicine. D.A. Gadd was funded by the Wellcome Trust 4-Year PhD in Translational Neuroscience—Training the Next Generation of Basic Neuroscientists to Embrace Clinical Research (108890/Z/15/Z). Drs Bernabeu and Marioni were supported by the Alzheimer’s Society major project grant AS-PG-19b-010. Generation Scotland (GS) received core support from the Chief Scientist Office of the Scottish Government Health Directorates (CZD/16/6) and the Scottish Funding Council (HR03006). Genotyping and DNA methylation profiling of the GS samples were performed by the Genetics Core Laboratory at the Clinical Research Facility, The University of Edinburgh, United Kingdom, and was funded by the Medical Research Council UK and the Wellcome Trust (references 104036/Z/14/Z, 220857/Z/20/Z, and 216767/Z/19/Z). The DNA methylation profiling and analysis were supported by Wellcome Investigator Award 220857/Z/20/Z and grant 104036/Z/14/Z (principal investigator: AM McIntosh) and through funding from National Alliance for Research on Schizophrenia & Depression (reference 27404; awardee: Dr D.M. Howard) and the Royal College of Physicians of Edinburgh (Sim Fellowship; awardee: Dr H.C. Whalley). All components of GS:SFHS (Generation Scotland: the Scottish Family Health Study), including the protocol and written study materials, have received formal and national ethical approval from the NHS Tayside Research Ethics Committee (Research Ethics Committee reference number 05/S1401/89). In addition, local approval has been obtained from the NHS Glasgow Research Ethics Committee and the NHS Glasgow and NHS Tayside Research and Development Offices, as required.

### Disclosures

L. Murphy and Dr Marioni received a speaker fee from Illumina. Dr Marioni is an advisor to the Epigenetic Clock Development Foundation. D.A. Gadd and Dr Marioni received consultancy fees from Optima Partners. The other authors report no conflicts.

### Supplemental Material

Supplemental Methods

Tables S1–S7

Figures S1–S3

References 36–46

## Supplementary Material
