# Supplementary material for: Epigenetic Contributions to Clinical Risk Prediction of Cardiovascular Disease
Source: Circ Genom Precis Med. Author manuscript; Available in PMC 2024 Mar 5. (PMC10876178; doi:10.1161/CIRCGEN.123.004265)
Supplement: 004265 - Acknowledgment Consent [file EMS193470-supplement-004265___Acknowledgment_Consent.pdf]

Circ

From: Robert Hillary <robert.hillary@ed.ac.uk>
Sent: Thursday, November 16, 2023 8:29 AM
To: Circ
Cc: Aleksandra Chybowska
Subject: CIRCCVG/2023/004265-T2: Consent for Acknowledgement

***** CAUTION: This email originated from outside of the American Heart Association. *****

Do **not** click links or open attachments unless you recognize the sender and know the content is safe.

Report Suspicious

Hello,

I am writing to confirm my consent to be acknowledged in the manuscript with reference number CIRCCVG/2023/004265-T2. Please let me know if there is anything else I can provide at this time.

Many thanks,
Robert Hillary

The University of Edinburgh is a charitable body, registered in Scotland, with registration number SC005336. Is e buidheann carthannais a th' ann an Oilthigh Dhùn Èideann, clàraichte an Alba, àireamh clàraidh SC005336.
