# Supplementary material for: Epigenetic Contributions to Clinical Risk Prediction of Cardiovascular Disease
Source: Circ Genom Precis Med. Author manuscript; Available in PMC 2024 Mar 5. (PMC10876178; doi:10.1161/CIRCGEN.123.004265)
Supplement: 004265 - Supplemental Material [file EMS193470-supplement-004265___Supplemental_Material.pdf]

SUPPLEMENTAL MATERIAL

Supplemental Methods

Generation Scotland (GS)

Generation Scotland is a population-based and family-structured cohort study of individuals from Scotland [36]. Between 2006 and 2010, patients at collaborating general medical practices in Scotland aged 35-65 years were invited to join the study. Subsequently, participants were asked to identify first-degree relatives aged 18 and over who were then invited to participate. 24,088 participants, aged 18-99 years, completed a health survey. Clinical and physical characteristics of 21,521 individuals who attended a clinic were measured using a standardized protocol. Fasting blood samples were obtained in clinic using a standard operating procedure.

Measurement of high-sensitivity troponin

The concentrations of high sensitivity cTnT and cTnI (Abbott Diagnostics) were measured in 19,130 GS individuals. Before the assay, samples were spun for 5 minutes at 2000g. The measurements were taken using i1000SR and Cobas e411 devices using the manufacturers' quality controls and calibrators. The limit of detection set by manufacturers of these devices is 1.2 ng/L. Anything below this limit is reported as a blank value. Correcting for blank values consisted of reporting results below the threshold of blank as 0.6 ng/L [8]. Initially reported in Welsh *et al.*, the coefficients of variation (CVs) for cTnT were 5.0% for the low control and 3.4% for the high control. CVs for cTnI were 6.2% for the low control, 6.0% for intermediate control, and 4.6% for high control [37].

DNA Methylation

DNAm was profiled in blood samples using the Illumina EPIC array. Quality control details have been described previously [38]. Filtering for poorly detected probes and samples, non-blood samples (e.g., samples obtained from saliva), and outliers was performed. Subsequently, non-CpG probes as well as probes on the X and Y chromosomes were removed. Missing CpG values were mean-imputed. To ensure any signatures generalise to as many cohorts as possible, the sites were subset to those also present on the older Illumina 450k array (n=453,093 CpGs). The full quality-controlled dataset contained 18,413 individuals.

Methylation profiling was carried out in three sets. Set 1 contained 5,087 individuals, Set 2 contained 4,450 individuals and Set 3 contained 8,876 individuals. Participants in Set 2 were genetically unrelated to each other and to those in Set 1 (genetic relationship matrix (GRM) threshold <0.05) while more complex relationship structures were present within and between Sets 1 and 3.

EpiScores for cardiac troponin

We generated an EpiScore for cTnI. The training set consisted of Sets 2 and 3 combined, whereas Set 1 was the test set. To minimise information from shared environments and relatedness leaking between the training and test sets, any individuals in the training set associated with the same family ID as individuals from the test set were excluded from further analyses. After this step, there were 9,754 individuals in the training set and 5,003 individuals in the test set.

As part of data pre-processing, all variables were trimmed of outliers (points beyond 3 SDs of the mean). The measured concentrations of cTnI underwent rank-based inverse normal transformation. Using linear mixed effects models, age, age^2 and sex were regressed out of the transformed variable, with a pedigree-based kinship matrix fitted as a random effect. The subsequent model residuals were saved and entered as the outcome of elastic net regression models. Methylation levels were treated as the independent variables. Elastic net models were run using biglasso (version 1.5.0) in R (version 4.0.3). The L1-L2 mixing parameter was set to alpha=0.5, and ten-fold cross-validation was applied. Non-zero coefficients/weights from the models were extracted. To calculate the EpiScore, CpG methylation values were multiplied by these coefficients and summed for each individual in the test set. The proportion of variance explained by the EpiScore was estimated by comparing R^2 estimates from the null and full linear regression models. Null models were adjusted for age, sex, and cTnI whereas full models accounted for age, sex, cTnI and the EpiScore.

EpiScores for 109 circulating proteins

We have previously generated 109 EpiScores for plasma protein levels [20]. Briefly, elastic net penalised regression models were run with 953 possible protein levels as outcomes and up to 428,489 DNAm measurements from the Illumina 450k array as input features. Protein levels were adjusted for genetic effects (protein quantitative trait loci – pQTLs) prior to training EpiScores. Protein EpiScores were trained in two cohorts: the Lothian Birth Cohort of 1936 (training set contained between 725 and 875 individuals for 160 Olink inflammatory and neurology proteins) and the German cohort, KORA (944 individuals, 793 SOMAscan proteins). 109 scores (84 trained in KORA and 25 trained in the Lothian Birth Cohort 1936) explained between 1% and 58% of the variance in protein levels (with $r>0.1$ and $P<0.05$) in independent test cohorts. In this study, the 109 protein EpiScores were projected into

Generation Scotland (n = 18,413) through the publicly available MethylDetectR Shiny App. A short tutorial on MethylDetectR is available at https://youtu.be/65Y2Rv-4tPU. Before applying weights, DNAm level at each site was scaled to have a unit SD and mean of zero. This procedure was performed separately in each set. The weighted combination of CpG sites for each protein EpiScore are identical to those used in the Gadd *et al*. study.

ASSIGN and SCORE2 scores

ASSIGN scores were calculated for 16,366 GS individuals with complete information about the component parts (age, sex, smoking status, systolic blood pressure, total cholesterol, high density lipoprotein cholesterol, family history of premature CVD, diagnosis of rheumatoid arthritis, diagnosis of diabetes, and a deprivation score). Variables were adjusted as per official guidance [39]. This included modifying the number of cigarettes smoked per day in recent quitters, adding 20mmHg to systolic blood pressure in individuals on blood pressure medications, and adding 10 to the number of cigarettes smoked per day in rheumatoid arthritis patients. The ASSIGN score was calculated in R using publicly available coefficients and formulae [40]. A subset of the obtained scores was validated against the scores produced by the online tool [21].

In addition to ASSIGN, which is tailored to the Scottish population, we also considered a European-wide risk measure, SCORE2, in sensitivity analyses. SCORE2 is a parsimonious predictor that utilises weighted information on age, sex, systolic blood pressure, cholesterol (HDL, LDL and total) and self-reported smoking (binary – yes/no). SCORE2 values were calculated for 16,934 GS individuals using external coefficients [41]. The estimates were calibrated according to region-specific scaling factors and validated against the online tool [42]. In a sample of individuals within the recommended SCORE2 age range (40-69 years old,

n=11,348), the Spearman correlation between of ASSIGN and SCORE2 was 0.89 (**Supplemental Figure I**). Given that the ASSIGN score was tailored to the Scottish population, we decided to use it as the primary CVD risk measure, with SCORE2 used in sensitivity analyses. The R scripts used to derive ASSIGN and SCORE2 are available at https://github.com/aleksandra-chybowska/troponin_episcores.

Cardiovascular disease events

CVD cases were ascertained through data linkage to NHS Scotland hospital records. Participants were followed to the end of September 2021. A composite CVD outcome was defined as per Welsh *et al.* [8] and included the following *International Classification of Diseases, 10th Revision codes*: I20–25, G45, I60–69, I00–I99, L29.5, L31.1, K40–46, K49, K75. A total of 2,265 incident CVD cases were observed over a follow up period of up to 16 years **(Supplemental Figure II).**

Statistical analysis for risk associations

Cox proportional hazards (PH) models were used to investigate the relationship between the CVD risk and potential multiomic biomarkers over 16 years of follow up. The studied biomarkers included: measured cardiac troponin, troponin EpiScores (see **Methods - EpiScores for cardiac troponin** for details), and 109 protein EpiScores. All models were adjusted for ASSIGN. Protein EpiScore models were additionally adjusted for the corrected concentration of cTnI (see **Methods - Measurement of high-sensitivity troponin)**. The levels of protein EpiScores as well as troponin concentrations were rank-based inverse normalised prior to the analyses. CVD cases comprised individuals diagnosed after baseline who subsequently died as well as of those who remained alive after receiving a diagnosis. Controls were censored at the end of the follow up period (September 2021) or at time of death (CVD-

free survival). Models based on data from merged Sets 1, 2, and 3 were generated using coxme library (v 2.2.16) with a kinship matrix fitted as a random effect to adjust for relatedness. The cox.zph function from the survival library (v. 3.2.7) [43] was used to examine Schoenfeld residuals (local and global Cox model assumptions tests with significance threshold $P < 0.05$). These models were not adjusted for relatedness. Survival, forest, and hazard-over-time plots were generated using a custom shiny application available at https://shiny.igc.ed.ac.uk/3d2c8245001b4e67875ddf2ee3fcbad2/.

Composite CVD EpiScore

An epigenetics-based CVD score referred to as the CVD EpiScore was generated. It considered the 109 DNAm scores reflecting the concentrations of plasma proteins (protein EpiScores) from Gadd *et al* [20] as potentially informative features ($n_{training}$=6,880, n_{test}=3,659, **Supplemental Figure III**). Whereas the weights used to calculate each individual protein EpiScore were taken from the Gadd *et al*. study, the weights applied to each EpiScore in the composite CVD EpiScore are novel to this study.

The score was derived using two different modelling techniques: Cox PH Elastic Net and Random Survival Forest. While Elastic Net models were trained using glmnet (v 4.0.2) [44], Random Survival Forest models were trained using randomForestSRC (v 3.2.0). Protein EpiScores were rank-based inverse normalised prior to training. To ensure that there were no individuals with overlapping family ids across the training and the test set, any individuals in the training set associated with the same family id as individuals from the test set were excluded from further analyses.

Elastic Net models were trained with the L1-L2 mixing parameter set to alpha=0.5 and with ten-fold cross-validation. The features with non-zero coefficients were used to generate composite score. Random Forest models were trained using ten-fold cross-validation. The 10-year onset probabilities were calculated and used as a composite CVD EpiScore.

Finally, to test whether the CVD EpiScore improved prediction of CVD over and above ASSIGN, the following Cox PH models were fit to the test set: a null model adjusted for age, sex, and ASSIGN, then full models, which included age, sex, ASSIGN score and 1) the CVD EpiScore, 2) the concentration of rank-based inverse normalised cTnI and 3) the CVD EpiScore + the concentration of cTnI.

Evaluating Performance of the Composite Scores

Cox PH analysis can be used to model the risk of incident CVD for different prediction periods. As the ASSIGN score estimates the risk of developing CVD over 10 years, we used 10-year CVD incidence to evaluate the classification performance of our models. To calculate the binary CVD outcome, time-to-event was truncated at 10-years. Prediction probabilities were obtained in the test set for each model by calculating the cumulative baseline hazard using the Breslow estimator [45]. Prediction metrics (Area Under the receiver operator characteristic Curve (AUC), Precision-Recall AUC and C-index) were then generated using the pROC package [46].

Supplemental Tables

Supplemental Table I. Hazard ratio (HR) of ASSIGN estimated by Cox proportional hazards CVD model adjusted for relatedness using kinship matrix where the ASSIGN score is the only predictor.

Age category	HR_{ASSIGN}	95% CI	P_{ASSIGN}	n_{cases}	$n_{controls}$
<30	2.42	[1.43, 4.10]	9.4×10^{-4}	20	2560
30 - 40	1.87	[1.48, 2.37]	2.0×10^{-7}	64	2436
40 - 50	1.97	[1.74, 2.22]	$<2.2 \times 10^{-16}$	232	3153
50 - 60	1.67	[1.55, 1.81]	$<2.2 \times 10^{-16}$	526	3763
60 - 70	1.54	[1.42, 1.67]	$<2.2 \times 10^{-16}$	530	2267
≥70	1.27	[1.12, 1.44]	2.3×10^{-4}	252	551

Supplemental Table II. Description of the 109 proteins for which Gadd et al. generated robust EpiScores. Table adapted from Gadd *et. al* [20].

Identifier (SOMAscan SeqId or Olink name)	***Protein Panel***	***Gene Name***	***Target***
4407-10	SomaScan	MST1	MSP
4435-66	SomaScan	ENPP7	ENPP7
4148-49	SomaScan	PAPPA	PAPP-A
3617-80	SomaScan	HGFAC	HGFA
3440-7	SomaScan	GZMA	granzyme A
3403-1	SomaScan	TPSB2	TPSB2
3291-30	SomaScan	FCER2	CD23
3195-50	SomaScan	GNLY	Granulysin
5124-69	SomaScan	ICAM5	sICAM-5
3216-2	SomaScan	PIGR	PIGR
5339-49	SomaScan	S100A9	calgranulin B
3175-51	SomaScan	ADAMTS13	ATS13
3311-27	SomaScan	FCGR3B	FCG3B
4500-50	SomaScan	CLEC11A	SCGF-alpha
2948-58	SomaScan	GHR	Growth hormone receptor
2950-57	SomaScan	IGFBP4	IGFBP-4
2966-65	SomaScan	CLEC11A	SCGF-beta
4337-49	SomaScan	CRP	CRP
2579-17	SomaScan	MMP9	MMP-9
4498-62	SomaScan	NCAM1	NCAM-120
3184-25	SomaScan	F7	Coagulation Factor VII
3324-51	SomaScan	LY9	LY9
5034-79	SomaScan	PRSS2	Trypsin 2
3343-1	SomaScan	ACY1	Aminoacylase-1
5358-3	SomaScan	OMD	OMD
3046-31	SomaScan	RETN	resistin
4568-17	SomaScan	SLITRK5	SLIK5
3448-13	SomaScan	INSR	IR
5028-59	SomaScan	CD163	sCD163
2658-27	SomaScan	NTRK3	TrkC
3292-75	SomaScan	CD48	CD48
4929-55	SomaScan	SHBG	SHBG
4924-32	SomaScan	MMP1	MMP-1
2580-83	SomaScan	MPO	Myeloperoxidase
4831-4	SomaScan	SELL	sL-Selectin

3152-57	SomaScan	TNFRSF1B	TNF sR-II
2967-8	SomaScan	VCAM1	VCAM-1
5000-52	SomaScan	LGALS3BP	LG3BP
3038-9	SomaScan	CXCL11	I-TAC
3169-70	SomaScan	IDUA	IDUA
3060-43	SomaScan	C9	C9
2665-26	SomaScan	TNFRSF17	BCMA
2516-57	SomaScan	CCL21	6Ckine
2780-35	SomaScan	LTF	Lactoferrin
4763-31	SomaScan	AFM	Afamin
3485-28	SomaScan	B2M	b2-Microglobulin
2771-35	SomaScan	IGFBP1	IGFBP-1
4481-34	SomaScan	C4A|C4B	C4
3505-6	SomaScan	LTA|LTB	Lymphotoxin a1/b2
3041-55	SomaScan	MRC2	MRC2
3470-1	SomaScan	SELE	sE-Selectin
3554-24	SomaScan	ADIPOQ	Adiponectin
4990-87	SomaScan	GP1BA	GP1BA
4920-10	SomaScan	LYZ	Lysozyme
2851-63	SomaScan	C5	C5a
3293-2	SomaScan	CD5L	CD5L
4496-60	SomaScan	MMP12	MMP-12
2687-2	SomaScan	MIA	MIA
3316-58	SomaScan	SERPIND1	Heparin cofactor II
3473-78	SomaScan	MPL	Thrombopoietin Receptor
2982-82	SomaScan	LGALS4	Galectin-4
3298-52	SomaScan	CNTN4	Contactin-4
3044-3	SomaScan	CCL18	PARC
5107-7	SomaScan	NOTCH1	Notch 1
4153-11	SomaScan	SERPINA3	alpha-1-antichymotrypsin complex
3805-16	SomaScan	ESM1	Endocan
3339-33	SomaScan	THBS2	TSP2
4160-49	SomaScan	MMP2	MMP-2
3079-62	SomaScan	RARRES2	TIG2
3519-3	SomaScan	CCL17	TARC
3035-80	SomaScan	IL19	IL-19
5363-51	SomaScan	SEMA3E	Semaphorin 3E
3235-50	SomaScan	WFIKKN2	WFKN2
2705-5	SomaScan	CCL25	TECK
3600-2	SomaScan	CHIT1	Chitotriosidase-1

4141-79	SomaScan	CXCL10	IP-10
3348-49	SomaScan	BMP1	BMP-1
3029-52	SomaScan	CD209	DC-SIGN
3508-78	SomaScan	CCL22	MDC
2816-50	SomaScan	BCAM	BCAM
2826-53	SomaScan	EDA	EDA
5491-12	SomaScan	SPOCK2	Testican-2
5029-3	SomaScan	FAP	SEPR
4930-21	SomaScan	STC1	Stanniocalcin-1
CRTAM	Olink Neurology	CRTAM	N/A
EZR	Olink Neurology	EZR	N/A
FcRL2	Olink Neurology	FCRL2	N/A
G.CSF	Olink Neurology	CSF3	N/A
GDF.8	Olink Neurology	MSTN	N/A
GZMA	Olink Neurology	GZMA	N/A
NEP	Olink Neurology	MME	N/A
N.CDase	Olink Neurology	ASAH2	N/A
NMNAT1	Olink Neurology	NMNAT1	N/A
NTRK3	Olink Neurology	NTRK3	N/A
SKR3	Olink Neurology	ACVRL1	N/A
SIGLEC1	Olink Neurology	SIGLEC1	N/A
SMPD1	Olink Neurology	SMPD1	N/A
CCL11	Olink Inflammatory	CCL11	N/A
CD6	Olink Inflammatory	CD6	N/A
CXCL10	Olink Inflammatory	CXCL10	N/A
CXCL11	Olink Inflammatory	CXCL11	N/A
CXCL9	Olink Inflammatory	CXCL9	N/A
EN.RAGE	Olink Inflammatory	S100A12	N/A
TGF.alpha	Olink Inflammatory	TGFA	N/A
FGF.21	Olink Inflammatory	FGF21	N/A
HGF	Olink Inflammatory	HGF	N/A
OSM	Olink Inflammatory	OSM	N/A
VEGFA	Olink Inflammatory	VEGFA	N/A
MMP.1	Olink Inflammatory	MMP1	N/A

Supplemental Table III. Hazard ratios associated with protein EpiScores included in three different models of cardiovascular disease (n=12,657, n_{events}=1,274). Models were adjusted for the following covariates: A – ASSIGN, AT – ASSIGN and the concentration of cardiac troponin I, ATC – ASSIGN, the concentration of cardiac troponin I and cell composition. Underlined P-values represent the EpiScores that violate the proportional hazards assumption of Cox regression.

ID	*Gene*	HR_A	P_A	HR_{AT}	P_{AT}	HR_{ATC}	P_{ATC}
2516-57	CCL21	1.10 [1.04, 1.16]	0.001	1.07 [1.01, 1.14]	0.015	1.08 [1.02, 1.15]	0.006
2579-17	MMP9	1.12 [1.06, 1.19]	<0.001	1.13 [1.07, 1.19]	<0.001	1.10 [1.02, 1.18]	0.014
2580-83	MPO	1.05 [0.99, 1.11]	0.094	1.05 [0.99, 1.11]	0.095	0.94 [0.86, 1.03]	0.180
2658-27	NTRK3	0.84 [0.79, 0.89]	<0.001	0.84 [0.79, 0.89]	<0.001	0.85 [0.79, 0.90]	<0.001
2665-26	TNFRSF17	0.99 [0.94, 1.04]	0.690	0.99 [0.94, 1.05]	0.770	1.01 [0.96, 1.07]	0.630
2687-2	MIA	0.90 [0.86, 0.95]	<0.001	0.91 [0.86, 0.96]	<0.001	0.93 [0.88, 0.98]	0.007
2705-5	CCL25	1.04 [0.99, 1.10]	0.140	1.04 [0.98, 1.10]	0.210	1.07 [1.01, 1.14]	<u>0.024</u>
2771-35	IGFBP1	0.90 [0.85, 0.95]	<0.001	0.92 [0.87, 0.97]	0.004	0.92 [0.87, 0.98]	0.007
2780-35	LTF	1.05 [0.99, 1.11]	0.085	1.04 [0.99, 1.10]	0.130	1.00 [0.94, 1.07]	0.950
2816-50	BCAM	1.03 [0.97, 1.09]	0.310	1.03 [0.97, 1.09]	0.320	1.03 [0.97, 1.08]	<u>0.380</u>
2826-53	EDA	0.97 [0.91, 1.02]	0.210	0.96 [0.91, 1.02]	0.170	0.95 [0.90, 1.01]	0.120

2851-63	C5	1.15 [1.09, 1.22]	<0.001	1.16 [1.09, 1.22]	<0.001	1.13 [1.06, 1.20]	<0.001
2948-58	GHR	1.04 [0.99, 1.10]	0.130	1.05 [0.99, 1.10]	0.100	1.11 [1.05, 1.18]	<0.001
2950-57	IGFBP4	1.20 [1.14, 1.27]	<0.001	1.20 [1.14, 1.27]	<0.001	1.19 [1.12, 1.26]	<0.001
2966-65	CLEC11A	1.07 [1.01, 1.13]	0.014	1.06 [1.00, 1.12]	0.045	1.01 [0.94, 1.08]	0.850
2967-8	VCAM1	1.10 [1.03, 1.17]	0.003	1.10 [1.03, 1.17]	0.003	1.11 [1.03, 1.18]	0.004
2982-82	LGALS4	1.07 [1.01, 1.14]	0.015	1.07 [1.01, 1.13]	0.026	1.05 [0.99, 1.11]	0.092
3029-52	CD209	0.91 [0.86, 0.97]	0.002	0.92 [0.87, 0.98]	0.006	0.96 [0.90, 1.02]	0.150
3035-80	IL19	0.97 [0.92, 1.03]	0.290	0.96 [0.91, 1.02]	0.190	1.00 [0.94, 1.07]	0.890
3038-9	CXCL11	1.04 [0.99, 1.10]	0.150	1.05 [1.00, 1.11]	0.068	1.04 [0.99, 1.10]	0.120
3041-55	MRC2	0.90 [0.85, 0.95]	<0.001	0.90 [0.85, 0.95]	<0.001	0.89 [0.80, 1.00]	0.045
3044-3	CCL18	1.21 [1.13, 1.29]	<0.001	1.19 [1.11, 1.27]	<0.001	1.17 [1.10, 1.26]	<0.001
3046-31	RETN	1.06 [1.01, 1.12]	0.030	1.06 [1.01, 1.12]	0.030	0.98 [0.89, 1.07]	0.620
3060-43	C9	0.99 [0.94, 1.05]	0.840	1.01 [0.95, 1.06]	0.830	0.92 [0.86, 0.99]	0.020
3079-62	RARRES2	1.20 [1.14, 1.28]	<0.001	1.21 [1.14, 1.28]	<0.001	1.22 [1.14, 1.30]	<0.001
3152-57	TNFRSF1B	1.09 [1.02, 1.15]	0.007	1.07 [1.01, 1.14]	0.021	1.04 [0.97, 1.11]	0.270
3169-70	IDUA	1.02 [0.97, 1.08]	0.390	1.02 [0.97, 1.08]	0.420	1.03 [0.98, 1.09]	0.280
3175-51	ADAMTS13	0.91 [0.86, 0.97]	0.002	0.92 [0.87, 0.97]	0.002	0.94 [0.89, 1.00]	0.044

3184-25	F7	0.98 [0.93, 1.03]	0.410	0.98 [0.93, 1.04]	0.580	0.98 [0.93, 1.04]	0.500
3195-50	GNLY	0.94 [0.89, 0.99]	0.018	0.95 [0.90, 1.00]	0.063	1.00 [0.93, 1.08]	1.000
3216-2	PIGR	1.12 [1.06, 1.18]	<0.001	1.13 [1.07, 1.19]	<0.001	1.12 [1.06, 1.18]	<0.001
3235-50	WFIKKN2	0.86 [0.81, 0.91]	<0.001	0.87 [0.82, 0.92]	<0.001	0.88 [0.83, 0.94]	<0.001
3291-30	FCER2	0.99 [0.93, 1.05]	0.710	0.98 [0.93, 1.04]	0.530	0.95 [0.88, 1.02]	0.150
3292-75	CD48	1.00 [0.94, 1.05]	0.870	1.00 [0.95, 1.06]	0.920	1.02 [0.96, 1.08]	0.510
3293-2	CD5L	1.12 [1.06, 1.18]	<0.001	1.12 [1.06, 1.18]	<0.001	1.12 [1.05, 1.19]	<0.001
3298-52	CNTN4	0.84 [0.80, 0.89]	<0.001	0.85 [0.80, 0.90]	<0.001	0.86 [0.81, 0.92]	<0.001
3311-27	FCGR3B	1.06 [1.00, 1.12]	0.037	1.06 [1.00, 1.12]	0.037	1.07 [1.01, 1.13]	0.027
3316-58	SERPIND1	1.07 [1.01, 1.13]	0.017	1.10 [1.04, 1.16]	0.001	1.11 [1.05, 1.18]	<0.001
3324-51	LY9	0.94 [0.89, 0.99]	0.022	0.95 [0.90, 1.01]	0.077	0.99 [0.93, 1.05]	0.760
3339-33	THBS2	1.12 [1.05, 1.18]	<0.001	1.12 [1.05, 1.18]	<0.001	1.10 [1.04, 1.17]	0.002
3343-1	ACY1	1.11 [1.05, 1.17]	<0.001	1.08 [1.02, 1.14]	0.010	1.10 [1.03, 1.16]	0.002
3348-49	BMP1	1.02 [0.97, 1.08]	0.490	1.00 [0.95, 1.05]	0.960	1.03 [0.97, 1.08]	0.380
3403-1	TPSB2	0.99 [0.94, 1.05]	0.760	0.99 [0.94, 1.04]	0.680	0.98 [0.93, 1.04]	0.500
3440-7	GZMA	1.00 [0.95, 1.06]	0.950	1.01 [0.95, 1.06]	0.810	1.10 [1.02, 1.19]	0.014
3448-13	INSR	0.96 [0.91, 1.01]	0.130	0.95 [0.90, 1.00]	0.063	0.97 [0.92, 1.03]	0.320

3470-1	SELE	1.18 [1.12, 1.25]	<0.001	1.16 [1.09, 1.22]	<0.001	1.14 [1.07, 1.21]	<0.001
3473-78	MPL	1.01 [0.96, 1.07]	0.730	1.01 [0.95, 1.06]	0.820	0.99 [0.93, 1.04]	0.650
3485-28	B2M	1.12 [1.05, 1.20]	<0.001	1.12 [1.05, 1.19]	<0.001	1.10 [1.03, 1.17]	0.006
3505-6	LTA|LTB	0.88 [0.83, 0.93]	<0.001	0.90 [0.85, 0.96]	<0.001	0.91 [0.85, 0.98]	0.011
3508-78	CCL22	1.04 [0.99, 1.10]	0.130	1.05 [1.00, 1.11]	0.063	1.04 [0.99, 1.10]	0.150
3519-3	CCL17	1.02 [0.96, 1.07]	0.560	1.02 [0.97, 1.08]	0.380	1.01 [0.96, 1.07]	0.690
3554-24	ADIPOQ	0.92 [0.88, 0.98]	0.005	0.95 [0.90, 1.01]	0.095	0.96 [0.90, 1.01]	0.110
3600-2	CHIT1	1.00 [0.94, 1.07]	0.940	0.99 [0.93, 1.06]	0.830	0.95 [0.89, 1.02]	0.180
3617-80	HGFAC	1.05 [0.99, 1.11]	0.098	1.05 [0.99, 1.11]	0.097	1.05 [0.99, 1.11]	0.100
3805-16	ESM1	1.00 [0.95, 1.06]	0.860	1.00 [0.95, 1.06]	0.980	0.99 [0.94, 1.05]	0.860
4141-79	CXCL10	1.05 [0.99, 1.11]	0.120	1.04 [0.98, 1.10]	0.170	1.03 [0.97, 1.09]	0.390
4148-49	PAPPA	1.02 [0.97, 1.08]	0.450	1.02 [0.96, 1.07]	0.580	1.00 [0.94, 1.06]	1.000
4153-11	SERPINA3	0.97 [0.92, 1.03]	0.340	0.99 [0.93, 1.04]	0.600	0.94 [0.88, 1.00]	0.034
4160-49	MMP2	0.87 [0.82, 0.92]	<0.001	0.87 [0.82, 0.92]	<0.001	0.87 [0.81, 0.93]	<0.001
4337-49	CRP	1.21 [1.15, 1.29]	<0.001	1.22 [1.16, 1.30]	<0.001	1.24 [1.16, 1.33]	<0.001
4407-10	MST1	1.03 [0.97, 1.08]	0.370	1.02 [0.97, 1.08]	0.380	1.02 [0.96, 1.08]	0.520
4435-66	ENPP7	1.12 [1.05, 1.18]	<0.001	1.11 [1.05, 1.17]	<0.001	1.11 [1.05, 1.17]	<0.001

4481-34	C4A|C4B	0.94 [0.89, 1.00]	0.036	0.95 [0.90, 1.00]	0.042	0.95 [0.90, 1.00]	0.055
4496-60	MMP12	1.13 [1.05, 1.22]	<0.001	1.13 [1.06, 1.22]	<0.001	1.11 [1.04, 1.20]	0.004
4498-62	NCAM1	0.88 [0.84, 0.93]	<0.001	0.87 [0.83, 0.92]	<0.001	0.88 [0.83, 0.93]	<0.001
4500-50	CLEC11A	1.06 [1.00, 1.12]	0.037	1.05 [0.99, 1.11]	0.094	1.00 [0.93, 1.07]	0.990
4568-17	SLITRK5	0.87 [0.83, 0.92]	<0.001	0.88 [0.84, 0.93]	<0.001	0.90 [0.85, 0.95]	<0.001
4763-31	AFM	1.04 [0.98, 1.10]	0.180	1.03 [0.98, 1.09]	0.250	1.15 [1.07, 1.23]	<0.001
4831-4	SELL	0.89 [0.83, 0.94]	<0.001	0.89 [0.84, 0.95]	<0.001	0.88 [0.83, 0.94]	<0.001
4920-10	LYZ	1.07 [1.01, 1.13]	0.020	1.06 [1.00, 1.12]	0.039	1.02 [0.96, 1.09]	0.470
4924-32	MMP1	1.14 [1.07, 1.20]	<0.001	1.14 [1.08, 1.21]	<0.001	1.12 [1.05, 1.19]	<0.001
4929-55	SHBG	0.89 [0.84, 0.94]	<0.001	0.91 [0.86, 0.97]	0.002	0.91 [0.85, 0.96]	<0.001
4930-21	STC1	1.16 [1.09, 1.23]	<0.001	1.12 [1.05, 1.20]	<0.001	1.10 [1.03, 1.18]	0.005
4990-87	GP1BA	0.97 [0.92, 1.02]	0.270	0.97 [0.92, 1.02]	0.230	0.95 [0.90, 1.01]	0.100
5000-52	LGALS3BP	1.09 [1.03, 1.15]	0.003	1.08 [1.02, 1.15]	0.006	1.09 [1.03, 1.15]	0.005
5028-59	CD163	1.05 [0.99, 1.11]	0.120	1.04 [0.98, 1.10]	0.170	1.05 [0.99, 1.12]	0.099
5029-3	FAP	0.93 [0.88, 0.98]	0.006	0.92 [0.88, 0.98]	0.005	0.95 [0.89, 1.02]	0.150
5034-79	PRSS2	1.17 [1.10, 1.24]	<0.001	1.15 [1.09, 1.22]	<0.001	1.16 [1.09, 1.23]	<0.001
5107-7	NOTCH1	0.83 [0.78, 0.88]	<0.001	0.84 [0.79, 0.89]	<0.001	0.82 [0.77, 0.88]	<0.001

5124-69	ICAM5	1.05 [0.99, 1.11]	0.100	1.06 [1.01, 1.12]	0.027	1.06 [1.01, 1.12]	0.030
5339-49	S100A9	1.14 [1.07, 1.20]	<0.001	1.13 [1.07, 1.20]	<0.001	1.27 [1.10, 1.46]	0.001
5358-3	OMD	0.86 [0.81, 0.91]	<0.001	0.87 [0.82, 0.92]	<0.001	0.88 [0.83, 0.93]	<0.001
5363-51	SEMA3E	0.90 [0.86, 0.95]	<0.001	0.89 [0.84, 0.94]	<0.001	0.89 [0.84, 0.94]	<0.001
5491-12	SPOCK2	0.94 [0.89, 1.00]	0.042	0.94 [0.89, 1.00]	0.039	0.98 [0.91, 1.05]	0.510
CCL11	CCL11	1.07 [1.01, 1.13]	0.026	1.08 [1.02, 1.14]	0.008	1.11 [1.05, 1.18]	<0.001
CD6	CD6	0.97 [0.91, 1.02]	0.210	0.97 [0.92, 1.03]	0.330	1.17 [1.05, 1.30]	0.005
CRTAM	CRTAM	1.03 [0.98, 1.09]	0.300	1.03 [0.98, 1.09]	0.220	1.09 [1.02, 1.17]	0.012
CXCL10	CXCL10	1.07 [1.01, 1.13]	0.020	1.06 [1.01, 1.13]	0.026	1.08 [1.02, 1.14]	0.009
CXCL11	CXCL11	1.00 [0.95, 1.06]	0.890	1.01 [0.95, 1.06]	0.790	0.99 [0.94, 1.05]	0.770
CXCL9	CXCL9	1.09 [1.03, 1.15]	0.003	1.10 [1.04, 1.16]	0.001	1.11 [1.05, 1.18]	<0.001
EN.RAGE	S100A12	1.07 [1.01, 1.13]	0.021	1.08 [1.02, 1.14]	0.009	1.05 [0.97, 1.14]	0.200
EZR	EZR	0.93 [0.88, 0.99]	0.013	0.93 [0.88, 0.99]	0.016	0.97 [0.89, 1.06]	0.450
FcRL2	FCRL2	0.92 [0.88, 0.98]	0.005	0.93 [0.88, 0.98]	0.007	0.93 [0.86, 1.01]	0.079
FGF.21	FGF21	1.17 [1.10, 1.24]	<0.001	1.18 [1.11, 1.25]	<0.001	1.23 [1.16, 1.31]	<0.001
G.CSF	CSF3	1.14 [1.08, 1.21]	<0.001	1.14 [1.08, 1.21]	<0.001	1.12 [1.05, 1.19]	<0.001
GDF.8	MSTN	0.94 [0.89, 1.00]	0.037	0.93 [0.88, 0.99]	0.014	0.94 [0.89, 1.00]	0.060

GZMA	GZMA	0.94 [0.89, 0.99]	0.023	0.94 [0.89, 0.99]	0.020	0.96 [0.88, 1.06]	0.440
HGF	HGF	1.17 [1.10, 1.23]	<0.001	1.18 [1.11, 1.25]	<0.001	1.18 [1.11, 1.26]	<0.001
MMP.1	MMP1	1.05 [0.99, 1.11]	0.098	1.05 [1.00, 1.11]	0.064	1.03 [0.97, 1.09]	0.390
N.CDase	ASAH2	0.99 [0.93, 1.04]	0.620	0.99 [0.93, 1.04]	0.660	1.01 [0.95, 1.07]	0.710
NEP	MME	1.11 [1.05, 1.17]	<0.001	1.10 [1.04, 1.17]	<0.001	1.18 [1.11, 1.25]	<0.001
NMNAT1	NMNAT1	0.98 [0.93, 1.03]	0.420	0.98 [0.93, 1.03]	0.480	0.97 [0.91, 1.03]	0.290
NTRK3	NTRK3	0.92 [0.87, 0.98]	0.005	0.91 [0.87, 0.97]	0.002	0.91 [0.86, 0.96]	<0.001
OSM	OSM	1.07 [1.01, 1.13]	0.016	1.08 [1.02, 1.14]	0.005	1.08 [1.00, 1.17]	0.065
SIGLEC1	SIGLEC1	1.11 [1.05, 1.18]	<0.001	1.12 [1.06, 1.19]	<0.001	1.13 [1.07, 1.20]	<0.001
SKR3	ACVRL1	1.14 [1.08, 1.21]	<0.001	1.15 [1.09, 1.22]	<0.001	1.13 [1.07, 1.20]	<0.001
SMPD1	SMPD1	1.06 [1.01, 1.12]	0.025	1.07 [1.01, 1.13]	0.021	1.07 [1.01, 1.13]	0.021
TGF.alpha	TGFA	1.10 [1.04, 1.16]	<0.001	1.12 [1.05, 1.18]	<0.001	1.11 [1.03, 1.19]	0.006
VEGFA	VEGFA	1.10 [1.04, 1.17]	<0.001	1.12 [1.06, 1.18]	<0.001	1.10 [1.04, 1.18]	0.002

Supplemental Table IV. Predictor weights for the 45 protein EpiScores included in the composite protein EpiScore.

Variable	*Coefficient*
GZMA	-0.1070275
N.CDase	-0.0013707
NTRK3	-0.0105284
SKR3	0.02292682
CCL11	0.01486593
CXCL11	-0.0363625
FGF.21	0.05884652
HGF	0.05611636
MMP.1	-0.0528479
2705-5	0.0411362
2851-63	0.07807877
2948-58	0.03807306
2967-8	0.08372915
3029-52	-0.0004437
3038-9	-0.0062101
3044-3	0.2568256
3060-43	-0.078839
3079-62	0.1024936
3152-57	0.01995523
3175-51	0.02976706
3291-30	0.03575731
3292-75	-0.0045541
3293-2	0.04389995
3339-33	0.04065941
3348-49	0.02393257
3403-1	-0.0070861
3448-13	0.02739967
3470-1	0.10485373
3485-28	0.05615907
3505-6	-0.0178471
3519-3	-0.0543479
3600-2	0.03783531
3617-80	0.03982561
4160-49	-0.0499596
4407-10	0.03860081

4435-66	0.01144093
4481-34	-0.0549405
4496-60	0.10710256
4763-31	0.03997106
4831-4	-0.0265954
4920-10	-8.29E-05
4924-32	0.0123399
5000-52	0.00608975
5124-69	0.00134476
5363-51	-0.0156491

Supplemental Table V. Summary of CVD risk models based on ASSIGN. N – null model (adjusted for age + sex + ASSIGN), E – CVD EpiScore, T – cardiac troponin I

Model	HR_{age}	P_{age}	HR_{sex}	P_{sex}	HR_{ASSIGN}	P_{ASSIGN}	HR_{E}	P_{E}	HR_{T}	P_{T}	AUC	PRAUC	C-index
**N**	1.01	0.54	1.13	0.23	2.05	$<2.2 \times 10^{-16}$	-	-	-	-	0.719	0.198	0.712
**N+E**	1.00	0.99	1.05	0.64	1.94	1.10×10^{-16}	1.3	5.6×10^{-3}	-	**-**	0.723	0.201	0.715
**N+T**	1.00	0.83	1.04	0.71	2.02	$<2.2 \times 10^{-16}$	-	**-**	1.16	1.1×10^{-2}	0.721	0.202	0.714
**N+E+T**	1.00	0.65	0.96	0.71	1.91	6.70×10^{-16}	1.32	3.7×10^{-3}	1.17	7.1×10^{-3}	0.724	0.205	0.717

Supplemental Table VI. Summary of CVD risk models based on ASSIGN. N – null model (adjusted for ASSIGN), E – CVD EpiScore, T – cardiac troponin I

Model	HR_{age}	P_{age}	HR_{sex}	P_{sex}	HR_{ASSIGN}	P_{ASSIGN}	HR_{E}	P_{E}	HR_{T}	P_{T}	AUC	PRAUC	C-index
**N**	-	-	-	-	2.15	$<2.2 \times 10^{-16}$	-	-	-	-	0.719	0.193	0.712
**N+E**	-	-	-	-	1.94	$<2.2 \times 10^{-16}$	1.31	2.7×10^{-3}	-	-	0.723	0.200	0.715
**N+T**	-	-	-	-	2.05	$<2.2 \times 10^{-16}$	-	-	1.17	5.0×10^{-3}	0.721	0.203	0.714
**N+E+T**	-	-	-	-	1.86	$<2.2 \times 10^{-16}$	1.30	3.9×10^{-3}	1.16	7.3×10^{-3}	0.724	0.205	0.716

Supplemental Table VII. Summary of CVD risk models based on SCORE2. N – null model (adjusted for age + sex + ASSIGN), E – CVD EpiScore, T – cardiac troponin I

Model	HR_{age}	P_{age}	HR_{sex}	P_{sex}	HR_{SCORE2}	P_{SCORE2}	HR_{E}	P_{E}	HR_{T}	P_{T}	AUC	PRAUC	C-index
**N**	1.02	0.05	0.96	0.75	1.79	$1.8x10^{-11}$	-	-	-	-	0.699	0.174	0.696
**N+E**	1.01	0.15	0.92	0.50	1.64	$5.4x10^{-8}$	1.34	$2.4x10^{-3}$	-	-	0.704	0.179	0.699
**N+T**	1.01	0.14	0.88	0.32	1.77	$6.3x10^{-11}$	-	-	1.18	$4.8x10^{-3}$	0.701	0.175	0.698
**N+E+T**	1.01	0.37	0.84	0.16	1.61	$1.8x10^{-7}$	1.36	$1.4\ x10^{-3}$	1.19	$2.7\ x10^{-3}$	0.707	0.182	0.702

Supplemental Figures

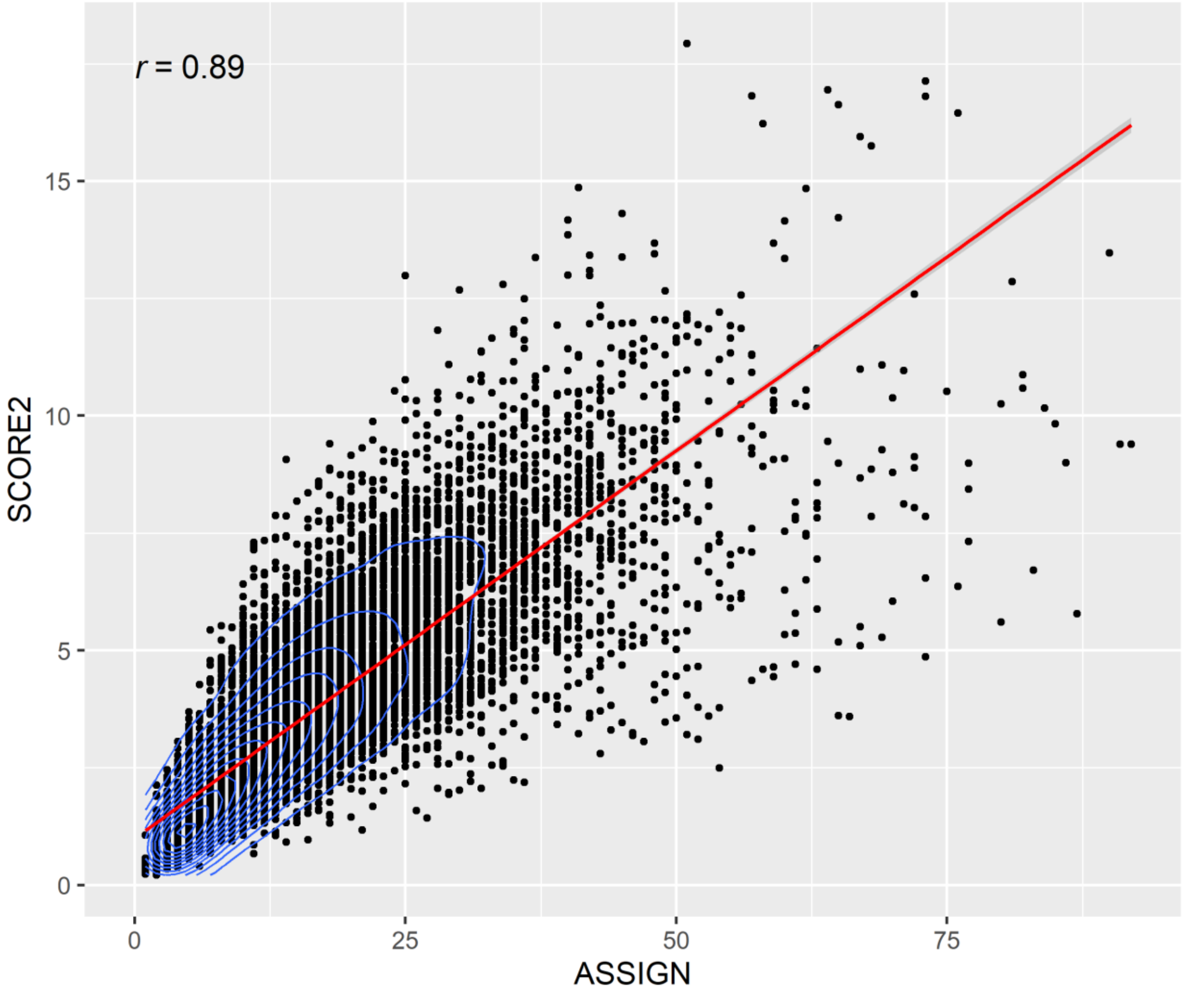

Supplemental Figure I. Strongly correlated 10-year CVD risk estimates produced by ASSIGN and SCORE2.

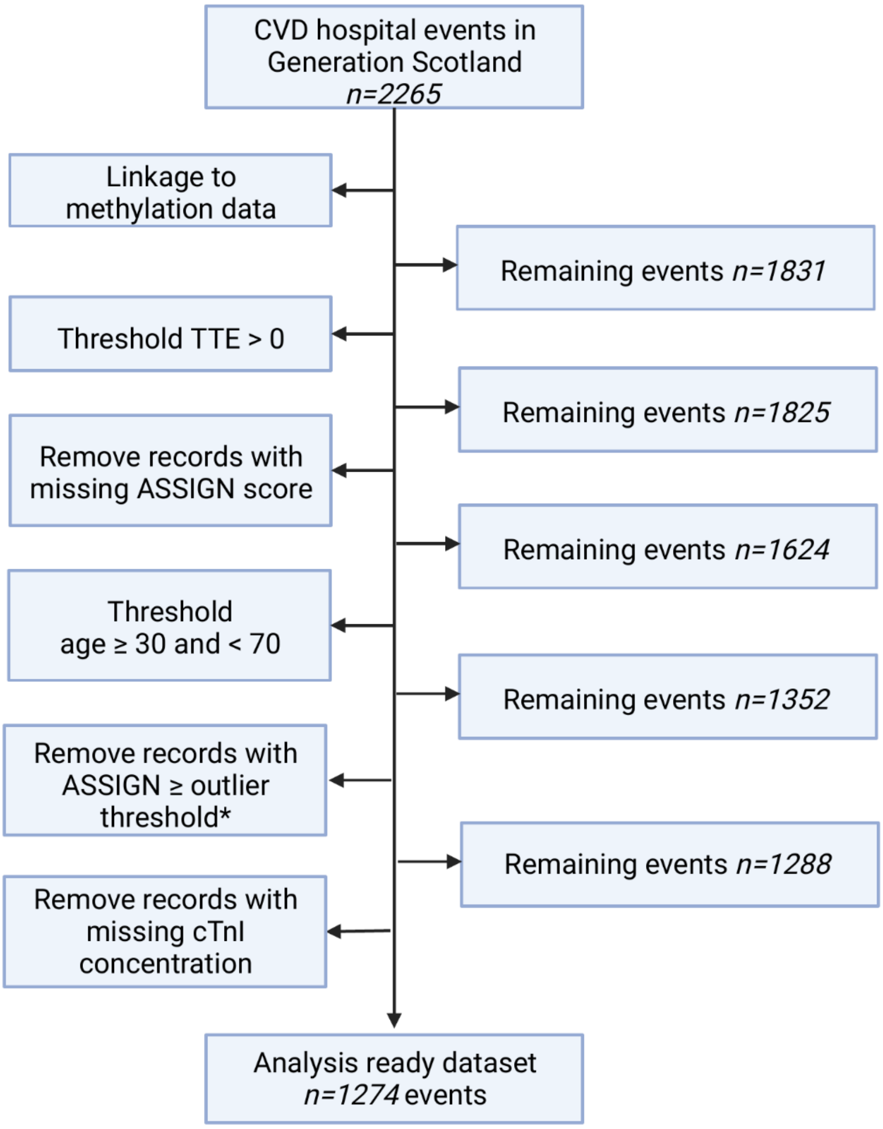

Supplemental Figure II. Pre-processing of the full dataset. This pipeline was used to prepare data for Cox PH models aimed at the identification of potential predictors of CVD. *Outlier threshold – an ASSIGN value that is more than 3 SD from the mean. Created with BioRender.com.

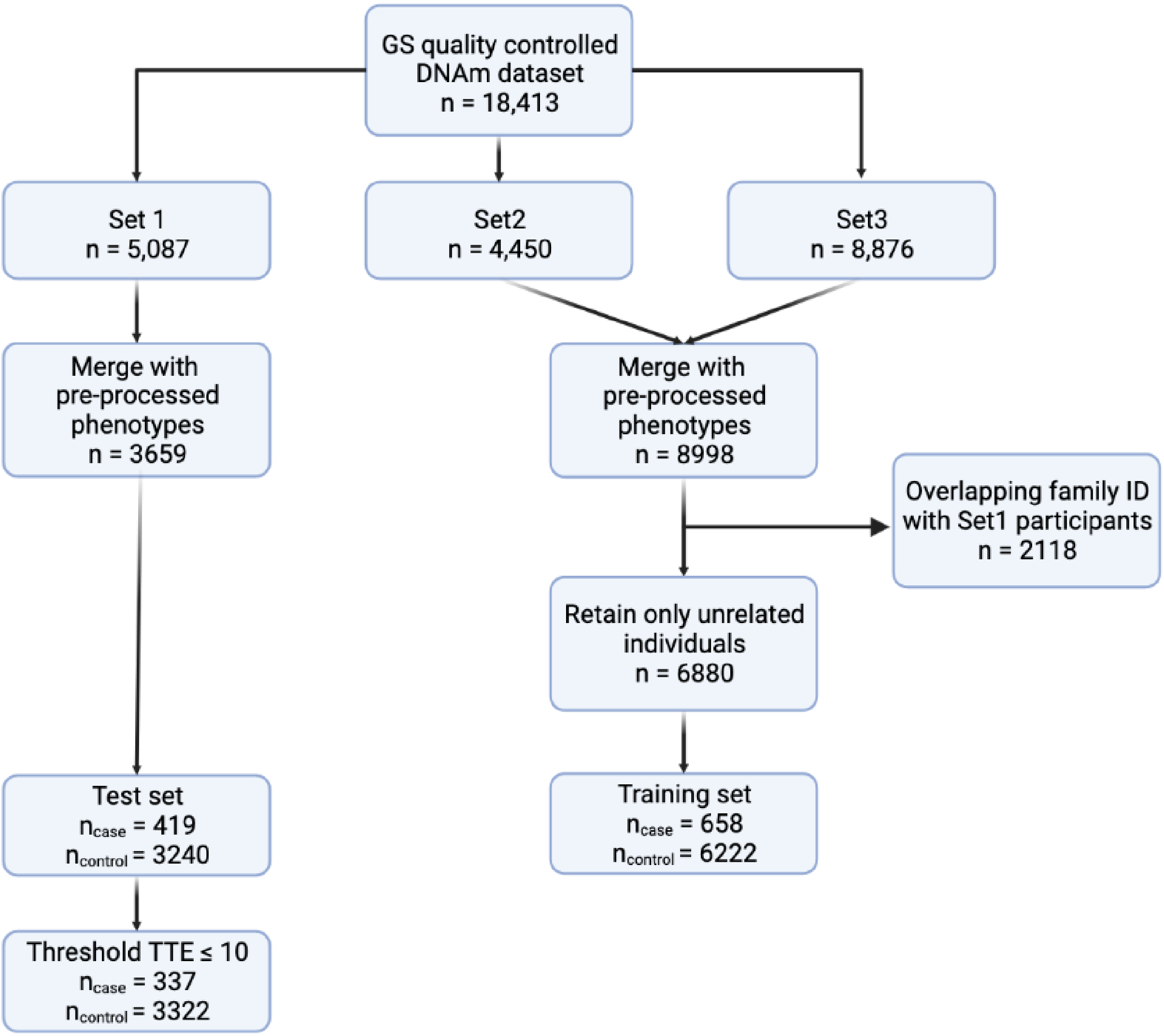

Supplemental Figure III. Pre-processing of the training and test sets. While Set 1 was the test set, the training set consisted of Sets 2 and 3 combined. Pre-processing phenotypes (ASSIGN components, composite CVD event status and time-to-event) consisted of filtering out records with time-to-event≤0, removing records with a missing ASSIGN score, thresholding age of participants to 30-70 years and removing outliers (ASSIGN value that is more than 3 SD from the mean). To ensure that there were no individuals with overlapping family ids across the training and the test set, any individuals in the training set associated with the same family id as individuals from the test set were excluded from further analyses. Created with BioRender.com.
